# Emerging Insights into Barriers to Effective Brain Tumor Therapeutics

**DOI:** 10.3389/fonc.2014.00126

**Published:** 2014-07-21

**Authors:** Graeme F. Woodworth, Gavin P. Dunn, Elizabeth A. Nance, Justin Hanes, Henry Brem

**Affiliations:** ^1^Department of Neurosurgery, University of Maryland School of Medicine, Baltimore, MD, USA; ^2^Department of Anatomy and Neurobiology, University of Maryland School of Medicine, Baltimore, MD, USA; ^3^Department of Neurosurgery, Pathology and Immunology, Center for Human Immunology and Immunotherapy Programs, Washington University School of Medicine, St. Louis, MO, USA; ^4^Center for Nanomedicine, Johns Hopkins University School of Medicine, Baltimore, MD, USA; ^5^Department of Ophthalmology, Johns Hopkins University School of Medicine, Baltimore, MD, USA; ^6^Department of Neurosurgery, Johns Hopkins University School of Medicine, Baltimore, MD, USA

**Keywords:** drug delivery, brain cancer, glioblastoma, nanotechnology, immunotherapy, advanced therapeutics, blood brain barrier, nanomedicine

## Abstract

There is great promise that ongoing advances in the delivery of therapeutics to the central nervous system (CNS) combined with rapidly expanding knowledge of brain tumor patho-biology will provide new, more effective therapies. Brain tumors that form from brain cells, as opposed to those that come from other parts of the body, rarely metastasize outside of the CNS. Instead, the tumor cells invade deep into the brain itself, causing disruption in brain circuits, blood vessel and blood flow changes, and tissue swelling. Patients with the most common and deadly form, glioblastoma (GBM) rarely live more than 2 years even with the most aggressive treatments and often with devastating neurological consequences. Current treatments include maximal safe surgical removal or biopsy followed by radiation and chemotherapy to address the residual tumor mass and invading tumor cells. However, delivering effective and sustained treatments to these invading cells without damaging healthy brain tissue is a major challenge and focus of the emerging fields of nanomedicine and viral and cell-based therapies. New treatment strategies, particularly those directed against the invasive component of this devastating CNS disease, are sorely needed. In this review, we (1) discuss the history and evolution of treatments for GBM, (2) define and explore three critical barriers to improving therapeutic delivery to invasive brain tumors, specifically, the *neuro-vascular unit* as it relates to the blood brain barrier, the *extra-cellular space* in regard to the brain penetration barrier, and the tumor *genetic heterogeneity and instability* in association with the treatment efficacy barrier, and (3) identify promising new therapeutic delivery approaches that have the potential to address these barriers and create sustained, meaningful efficacy against GBM.

## Challenges to Therapy for Infiltrating Brain Tumors – Defining the Problem

Brain cancer includes a diverse set of intracranial neoplasms and is the leading cause of cancer-related deaths in patients younger than 35 years (1, 2). Half of all *primary* brain tumors arise from cells within the brain (intrinsic lesions) while the remainder originate in the meninges or nerves (extrinsic lesions). The majority of primary intrinsic tumors arise from glial cells, hence the broad classification of these tumors as “gliomas.” The World Health Organization (WHO) has organized gliomas into a four-tiered histological grading scheme, where WHO Grade I (i.e., pilocytic astrocytoma) represents the more slow growing variant and WHO Grade IV [i.e., glioblastoma (GBM) multiforme] is the most malignant form characterized by cellular atypia, high mitotic index, neovascularization, and tissue necrosis. Malignant glioma (MG) traditionally encompasses WHO Grade III and IV lesions, since these tumors have a more aggressive growth pattern and are associated with a poor prognosis. Interestingly, MG is locally aggressive within the central nervous system (CNS), but very rarely metastasizes to other locations. The invasive tumor cells can be found far from the main tumor mass even in the more histologically benign forms (3). The importance of this characteristic is supported by the finding that tumor recurrence, even after apparent complete surgical resection by visual inspection and/or magnetic resonance imaging (MRI), causes significant neurological damage and eventual death from this disease (4).

Understanding the critical importance of residual invasive tumor cells, a neurosurgeon named Walter Dandy began removing the entire involved cerebral hemisphere in patients with suspected glioma (5). However, even with this aggressive surgical approach, his patients went on to succumb to tumor recurrence. Matsukado and colleagues analyzed the post-mortem brains of patients with gliomas and found tumor cells in the *contralateral* hemispheres in 50% of these patients (6). Hence, even with advanced surgical technologies, including stereotactic localization, intra-operative and functional MRI, real-time brain mapping, and fluorescence-guided surgery, the vexing problem of residual invasive cells within functional brain tissue still remains – surgery alone is unlikely to cure this disease.

**Figure 1:**
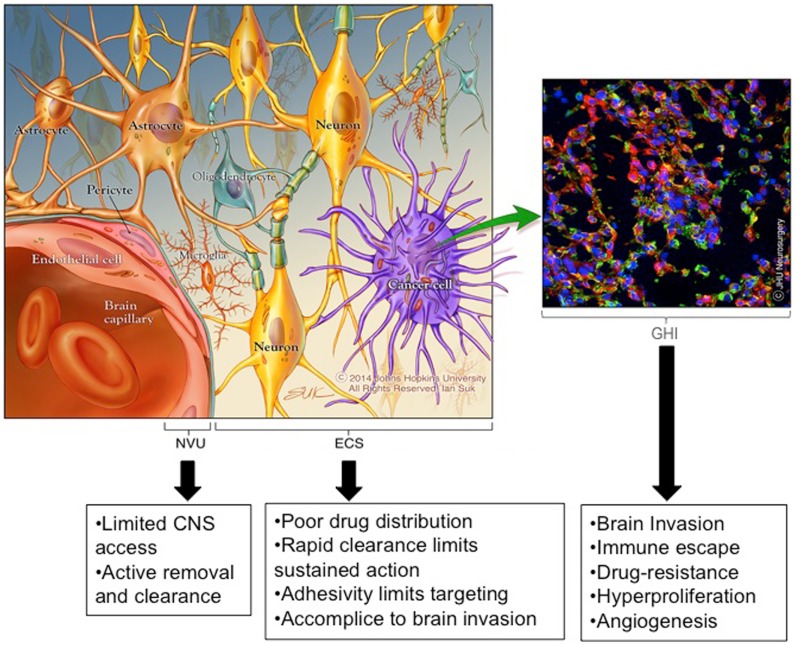
**Emerging insights into barriers to effective brain therapeutics**. Drug therapies used or designed for the treatment of invading glioma cells are hindered by three significant CNS and tumor-related physio-anatomic barriers: (1) the neuro-vascular unit (NVU) [related to the blood brain barrier (BBB)], which regulates the trafficking of substances between the blood stream and the brain, (2) the extra-cellular space (ECS) [related to the brain tissue/tumor penetration barrier], which comprises 15–20% of the total brain volume and affects the flow of nutrients, metabolites, cytokines, neurotransmitters, and numerous other molecules within tumors and brain tissue (the ECS components are not depicted to simplify the image), and (3) genetic heterogeneity and instability [related to the treatment effectiveness barrier], which enables the development of treatment resistant cells and redundant pathogenic mechanisms including immunologic escape, angiogenesis, hyperproliferation, invasion, and drug resistance. *Copyright Ian Suk 2014 – Johns Hopkins University.

The history of post-operative adjuvant therapies for glioma is one filled with attempts to deliver drugs to invading cancer cells while sparing the adjacent brain tissue. Drug therapies used or designed for this purpose are hindered by three significant brain- and tumor-related physio-anatomic barriers: (Figure 1): (1) the neuro-vascular unit (NVU) [related to the blood brain barrier (BBB)], which regulates the trafficking of substances between the blood stream and the CNS, (2) the extra-cellular space (ECS) (related to the brain tissue/tumor penetration barrier), which comprises 15–20% of the total brain volume and affects the flow of nutrients, metabolites, cytokines, neurotransmitters, and numerous other molecules within tumors and brain tissue, and (3) genetic heterogeneity and instability (related to the treatment efficacy barrier), which enables the development of treatment-resistant cells and redundant pathogenic mechanisms including immunologic escape, angiogenesis, hyperproliferation, invasion, and drug resistance.

### The neuro-vascular unit and blood brain barrier

The BBB is a unique biologic interface that separates the CNS from the rest of the body. Given the crucial role of the CNS in overall body function and health, the NVU has evolved to tightly regulate the exchange of most substances, including microbial, cellular, and metabolic elements. The NVU consists of a continuous layer of specialized endothelial cells linked together by tight junctions; this layer is supported by adhesions and interactions with basement membranes, brain pericytes, astrocytes, and neurons (Figure 1). While some small (<400 Da), relatively lipophilic molecules, can freely diffuse across the BBB, studies suggest that more than 90% of small molecules and nearly all large molecules are unable to passively cross this barrier (7–9). In one study exploring drugs used in the treatment of CNS diseases, the Comprehensive Medicinal Chemistry database of over 7000 available pharmaceuticals was queried and it was found that few of these drugs effectively cross the BBB (7).

In addition to size and physico-chemical restrictions, numerous active transporters exist to either increase or decrease the flux of substances across the BBB interface (10). Examples include glucose transporters (i.e., GLUT1) and molecular exporters (i.e., P-glycoproteins). Active molecular transporters add an additional complexity to the BBB on top of the stringent requirements for passive diffusion.

In certain disease processes, such as tumors, inflammation, and infection, the structure of the BBB is altered, leading to extravasation of a more varied group of substances into the associated brain tissue (11–13). The administration of intravenous “contrast” agents, which passively accumulate in these areas but not in unaffected regions, takes advantage of a disrupted BBB to aid in the diagnosis of some CNS conditions. In addition, the enhanced permeability and retention (EPR) effect (14) has been described for nanoparticulate delivery systems, where nanoparticle (NP) accumulation in neoplastic tissue is increased, likely due to increased movement of particles through wider fenestrations in the immature or malformed blood vessels, and NP clearance is decreased due to incomplete pseudo-lymphatic drainage pathways (15, 16). While the BBB is compromised in many gliomas, BBB breakdown is often heterogeneous throughout the tumor and generally remains intact in brain regions where infiltrating cells are found (17). Therefore, the BBB remains a key hurdle in the treatment of infiltrating gliomas. Strategies for crossing the BBB will be discussed later, and include: enhancing BBB permeability, using alternative routes such as intranasal, intrathecal, or local delivery, and employing targeting/shuttle systems to take advantage of endogenous transporters.

### The brain extra-cellular space and brain penetration barrier

While the BBB has long been considered the major barrier for therapeutic delivery within the CNS, more recently poor distribution of agents within the brain and/or tumor tissue itself has emerged as a major delivery challenge (18, 19). The ECS in brain tissue represents the major pathway for movement of many signaling molecules and metabolites, as well as therapeutic and diagnostic substances (20).

If a substance crosses the brain-related barriers into the parenchyma or is administered locally within the brain, it next encounters the space between cells called the “extracellular” or “interstitial” space. Movement in the ECS is governed by diffusion and bulk flow. Diffusion is the passive, random movement of substances that can occur either in relation to a concentration gradient, where there is a positive net flux of the substance within a medium toward regions of lower concentration, or without a concentration gradient where there is no net flux. Bulk flow is the movement of substances due to an energy or pressure gradient driving the motion of fluid and material through a space. This directional movement in the brain and tumor ECS is driven in part by the flow of interstitial fluid from higher to lower pressure as well as the significant contribution of arterial/brain pulsations to this fluid flow (21, 22). Critical to the discussion of intrinsic brain tumors are the interstitial pressure gradients commonly found within these tumors. Abnormally permeable tumor vasculature leads to fluid leakage from the intravascular space into the ECS, leading to the higher interstitial pressures found within tumors compared to the surrounding brain (23–25). The eventual distribution and retention of a given material in the brain is, therefore, related to its movement via diffusion and bulk flow, in combination with the relative rates of clearance, including degradation and partitioning into other spaces. Substance removal can occur by means of cell-mediated phagocytosis or uptake, enzymatic and/or chemical degradation, and passive or active transport into the blood, cerebrospinal fluid (CSF), or cells. In addition, the brain has been shown to have a “pseudo-lymphatic,” more recently termed “glia lymphatic or glymphatic,” drainage system, where cerebral extra-cellular fluids exchange with CSF and are removed either through the arachnoid villi into venous blood or via para-vascular and para-neural routes into lymph fluid (16, 26).

The brain ECS contains a complex network of lipids, polysaccharides, and proteins with electro-statically charged as well as hydrophobic regions. ECS volume shifts with changes in cerebral metabolic activity and blood flow (20, 21). Importantly, the ECS may be significantly altered in and around brain tumors, further increasing the challenge of movement within the ECS (27, 28). Vargova and colleagues found that the ECS volume fraction and complexity (also termed, “tortuosity”) both increase with tumor grade (27). Their study suggests that, contrary to the common conception of MG as a mainly hypercellular lesion, higher grade glial tumors also have a larger, more complex extra-cellular component, which is likely to contribute significantly to the patho-physiology of the disease. This idea is supported by numerous studies describing the link between the extra-cellular matrix structure and tumor invasion, recurrence, and patient survival (29–31). Herolde-Mende and colleagues correlated glioma grade and patient survival with the amount of a key ECS component (tenascin C) in MGs (31). Interestingly, tenascin proteins have been shown to enhance tumor cell proliferation and migration, and promote angiogenesis in gliomas (32–34). Sontheimer et al. showed that primary brain tumors exploit ion channels and transporters that serve to support homeostatic functions in normal brain tissue, enabling glioma cells to rapidly adjust their size and shape to climb through the small, sticky pores within extra-cellular brain spaces (35). Together, these data demonstrate the important link between the ECS and MG patho-physiology.

The physico-chemical properties, including mesh spacing, of the brain extra-cellular matrix are keys factors in the movement of materials within the brain. Previous studies have detailed the complex nature of the brain ECS, including electro-statically charged and hydrophobic areas, channel and dead space regions, and a virtual briar patch of matrix components including proteoglycans, glycosaminoglycans, and hyaluronic acid structures (20, 36–39). More closely defining the size limits and surface property characteristics required for movement within the brain ECS has greatly aided the establishment of design criteria for therapeutic and diagnostic delivery systems aimed at movement within the brain ECS (40). Effective ECS penetration by drug delivery systems will be important to enable dispersion of therapeutics or diagnostic agents and/or to allow cell- or structure-specific targeting in the CNS. Regardless of how the drug is delivered (oral, intravascular, CSF-mediated, or direct interstitial delivery), penetration of therapeutic agents to distant residual cells is crucial to the eventual efficacy of a treatment.

### Genetic heterogeneity and instability and treatment efficacy barrier

When detailing the critical physiologic and anatomic considerations for therapeutic delivery to infiltrating brain tumors, it becomes important to consider the complex, moving target these tumors represent. It is likely that what we consider histopathologic “MG” actually comprises a spectrum of molecularly heterogeneous diseases. Moreover, recent work has detailed the heterogeneity that exists within the tumors individual patients. Specifically, large-scale multi-platform profiling studies have revealed that there are roughly four subtypes of MG that are defined by differences in transcriptional signatures (41–43). Additionally, complementary copy number analysis and next generation sequencing approaches have pointed to the distinct molecular features that define each of these subtypes (44). The genetic subgroups include the classical [epidermal growth factor receptor (EGFR)-driven], proneural [platelet derived growth factor (PDGF)-driven], mesenchymal [neurofibromatous type I (NF1)-driven], and neural categories. With the proneural group, extensive work over the last 5 years has demonstrated that *IDH1*-mutant tumors exhibit strikingly distinct biological and clinical features (45–47). Thus, these studies have begun to describe the heterogeneity that exists within the MG histopathologic umbrella.

It is also likely that significant complexity exists within each individual tumor. Stommel et al. (48) showed that at least three receptor tyrosine kinases (RTKs) appear to be activated cooperatively within individual MG, suggesting that targeted RTK monotherapy will not be effective in treating tumors with multiple concomitant RTK drivers (48). The commonly expressed epidermal growth factor receptor variant III (EGFRvIII) variant of the EGFR receptor, which is an extra-cellular truncation of the wild-type receptor, is also known to have a heterogeneous distribution (49). A subset of this intratumoral complexity can be explained by clonal RTK genomic co-amplification. Roughly 10% of GBMs harbor amplifications of multiple RTKs such that tumors can be comprised of discrete cell populations each harboring amplification of a distinct RTK (50, 51). These data point to the idea that each tumor may be comprised of an admixture of distinct diseases and underline the challenges of targeting specificity.

An increasing number of studies have detailed the diverse gene expression profiles found in human gliomas and the numerous pathologic mechanisms involved, including immune escape, angiogenesis, hyperproliferation, invasion, and drug resistance (Figure 1) (29, 41, 43, 44, 46, 47, 52–57). Most of these studies compare the transcriptome or chromosomal changes found in different grades of glial tumors, which has led to an emerging genetic classification scheme (44, 52). In addition to genetic diversity, it is becoming clear that when selective pressure is placed on MG, the high propensity for genetic mutation and redundant pathogenic mechanisms enable the rapid emergence of clones that are resistant to the applied pressure (58). Genetic instability and pathogenic redundancy are evidenced by the numerous DNA repair and methylation mechanisms that are commonly mutated in primary brain cancers, including the well-studied genes encoding p53 and O6-methylguanine methyltransferase (MGMT) (46, 52, 58–61). An important example of the ramifications of genetic instability of glial tumors was observed in the Phase II trial of the EGFRvIII peptide vaccination. In this study, a significant percentage of patients elicited a specific antibody response to the EGFRvIII antigen, but at the time of tumor recurrence, 82% of the tumors had lost EGFRvIII expression (62). Hence, whether the “selective pressure” is a tumor-specific antibody, antigen-specific cytotoxic T-cell, chemotherapeutic drug, or selective small molecule inhibitor, resistant subpopulations of MG cells emerge to produce tumor recurrence particularly when targeting a single antigen or molecule.

Together the unique genetic sub-classifications and the inherent genetic instability of MG cells create the potential for vast clonal diversity. In addition, studies suggest there are also loco-regional differences in the cellular genetics, likely related to environmental changes experienced by the tumor cells in distinct tumor regions (63). This has led some to suggest that glioma cells may be viewed as two regional subtypes: (1) stationary proliferative cells generally found within the main tumor mass, and (2) migratory invasive cells located in more distant brain parenchyma. Importantly, these two cell populations have been shown to have quite different genetic profiles and active cellular pathways, and therefore may require distinct therapeutic targets and approaches (63).

In other cancers where genetic diversity and instability contribute significantly to disease pathogenesis, treatments that offer continuous, combined effects have proven to produce the most durable benefits (64–68). Sporadic or episodic treatments have been shown to allow the evolution of treatment resistance and lead to earlier disease progression when compared to sustained treatment strategies (69–71). Although MG has undergone some of the most extensive molecular classification across all cancer types, we have not yet been able to target particular driver mutations with the same level of success as has been observed in other settings such as in *BRAF*-mutant melanoma, *EGFR*-mutant lung cancer, or *HER2*-amplified breast cancer. A greater understanding of intratumoral genomic heterogeneity and instability potential will be critical to harnessing our molecular understanding of these diseases.

## Clinical Trials and the Standard of Care: Successes, Failures, and Lessons Learned

**Figure 2:**
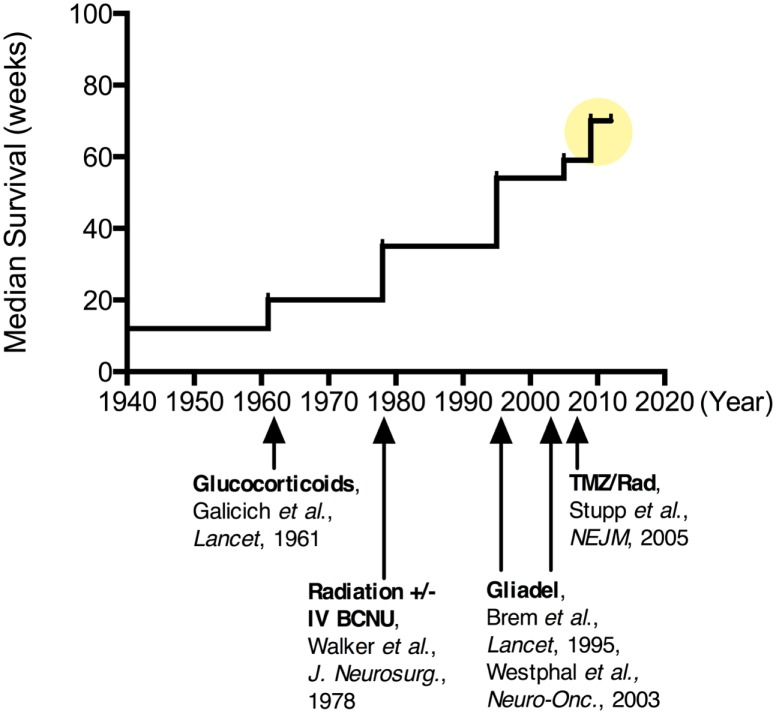
**Improvements in median survival over time for patients undergoing various treatments for malignant glioma**. Since the 1960s when corticosteroids were introduced for tumor-associated brain edema, there has been more than a quadrupling of the median survival for these patients (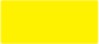). More recently, combination chemotherapy regimens have been suggested to increase this median survival upwards of 20 months.

Clinical research in treatments for MG has a rich history, with reports of hundreds of clinical trials of various types and approaches being published (72, 73). The vast number of research studies exploring treatment modalities for MG makes review and interpretation complex. However, insights can be gained by examining the evolution of the standard of care, with an emphasis on some of the key successes and failures over this time (Figure 2). Numerous early studies, including those dating back to the 1960s, were well-designed with appropriate controls, providing sound, evidence-based guidelines.

One of the first key discoveries came from the University of Minnesota in 1961 by Drs. Galicich, French, and Melby, who described the use of systemic corticosteroids (dexamethasone) to reduce peri-tumoral cerebral edema in patients with brain tumors (74). While this treatment was not evaluated on the basis of halting tumor progression or improving patient survival, it improved many of the neurological symptoms (weakness, aphasia, headache, and others) attributed to MG both before and after surgery (75). The major downside of this therapy was the side-effects of long-term (>2 weeks) steroid use including psychiatric changes, immunosuppression, osteopenia, skin remodeling, fat redistribution, and peptic ulcers. However, the profound improvements seen with this anti-inflammatory therapy continue to place corticosteroids in a central role in the management of tumor-associated edema for patients with MG. Not to be overlooked, the immunosuppressive and BBB modulating effects of dexamethasone (76–78) are also important in considering systemically administered or immunologic treatment strategies for MG patients in need of anti-edema therapy.

Also during the 1960s, radiation therapy (RT) in the form of whole-brain radiation began to emerge as an efficacious adjuvant therapy for MG (57). As with other cancers, the non-specific targeting of rapidly dividing cells by RT increased survival for many patients with MG (58, 59); RT typically doubled survival from about 6 to approximately 12 months. Whole-brain RT (WBRT) soon became the standard of care and characterized the control arm for future treatment studies (60). However, the maximum WBRT dose prescribed is limited by the radiation tolerance of critical CNS structures, such as the frontal lobes, optic apparatus, and brainstem. Alternative fractionation schemes and techniques, including dose escalation, hyper- and hypo-fractionation, brachytherapy, charged particles, and radiosensitizing drugs, have been explored, but none have consistently demonstrated improvement in survival. Eventually a regional, fractionated radiation approach was found to be as effective as WBRT, providing a high dose to a more focused region while minimizing toxicity (61, 62). Currently, most patients with MG receive intensity modulated radiation therapy (IMRT) fractionated in daily doses of 2 Gy given 5 days per week for 6 weeks, for a total radiation dose of 60 Gy (5).

With the roles of steroids and RT firmly in place, studies of chemotherapeutic drugs known as alkylating agents dominated the major clinical trials through the 1990s. In particular, carmustine (BCNU) and more recently, temozolomide (TMZ, oral formulation: Temodar), have been the focus of many chemotherapy studies for gliomas. A 2002 meta-analysis suggested that systemic administration of nitrosoureas, like BCNU, added approximately 2 months to the median survival for patients with high grade glioma (79). Despite this modest improvement, systemically administered BCNU was adopted into the standard of care at many centers through the mid-1990s.

In 1996, an implantable BCNU-loaded biodegradable polymer (Gliadel^®^), was approved by the FDA for the treatment of recurrent MG (Grade IV) (80). These drug-loaded interstitial wafers were designed to line the surgical resection cavity and deliver chemotherapy directly to residual tumor cells following MG surgery. Interstitial chemotherapy (IC) treatment consists of up to eight dime-size wafers made of a poly-anhydride biodegradable polymer impregnated with BCNU, providing sustained release of the drug over a 2–3-week period. IC therapy has shown the potential for local delivery to improve efficacy while reducing systemic side-effects, such as pulmonary fibrosis and myelosuppression, in the case of BCNU (81, 82). By 2004, Gliadel^®^ wafers were approved for all patients with primary and recurrent MG based on data from randomized controlled trials (80, 82, 83). This FDA approval marked a transition toward incorporating unique delivery strategies for MG and a broader recognition of the importance of mitigating the BBB in successful MG treatment approaches.

Since then, numerous studies and trials have explored local delivery approaches to take advantage of the unique drug delivery opportunity at the time of surgery. These have included regional and antibody-targeted brachytherapy, drug-loaded polymer and formulation strategies, and catheter-based infusions, with and without convection enhancement. To date, none of these approaches have shown a significant improvement in patient survival beyond standard therapies. A notable study, the PRECISE Trial, investigated the catheter-based, convection-enhanced delivery (CED) of an interleukin 13–*Pseudomonas* exotoxin fusion protein (IL-13–PE) compared to Gliadel in patients with recurrent MG. The IL-13–PE construct was designed to target glioma cells via the IL-13 receptor, and then deliver the potent bacterial toxin (PE) (84). Patients underwent tumor resection and were randomized to receive either two to four interparenchymal catheters with infusion over 4 days, or chemotherapy wafers (Gliadel) implanted at the time of surgery. Median survival did not differ significantly between the two groups (36.4 versus 35.3 weeks, *p* = 0.48) (85).

The PRECISE trial has been evaluated with regard to the limitations to effective therapy described above. First, the authors concluded that a major cause of limited efficacy was likely poor drug distribution or inaccurate catheter positioning based on *post hoc* analysis (86). This rationale highlights the importance of penetration within the brain in order to achieve an effective drug distribution, regardless of the pressure gradient (bulk flow) driving this process. Interestingly, limited and variable movement has been a consistent problem for numerous drugs and agents being studied with CED in the brain (87–90), likely due to physico-chemical interaction, partitioning effects, and/or degradation. Second, the potential for combination anti-tumor effects using the immuno-adjuvant properties of the IL-13 pathway and the potent toxicity profile of the *Pseudomonas* exotoxin created the potential for a powerful, multi-modal therapy (84). The one delivery-related component missing may have been the sustained action needed for a durable effect and, therefore, the need for infusions over 4 days. In the end, treatment modalities that address one, but not the other of these therapeutic limitations, will likely have marginal therapeutic efficacy.

Following the randomized studies on RT, systemic BCNU, and local BCNU (Gliadel wafers), a landmark study by Stupp et al. completed in 2005 established the current adjuvant therapy regimen for patients with MG (91). In this study, patients treated with surgery plus RT alone were compared to patients treated with this regimen plus the oral chemotherapy, TMZ. TMZ was given together with post-operative RT for 6 weeks, followed by intermittent doses over the following 6 months. Median survival with RT plus TMZ was significantly longer than with RT alone (14.6 versus 12.1 months). Perhaps more impressive, the 2-year survival for the control group was 10% compared to 26% in the TMZ group. These findings have created a paradigm shift in the clinical management of MG patients. The current, non-experimental treatment options for patients with MG shown to improve survival include: surgery, RT, oral chemotherapy (TMZ), and implantable chemotherapy (Gliadel).

In 2009, the FDA made a provisional approval of bevacizumab (Avastin) for patients with progressive MG failing standard therapy. The approval was based on the results of well-designed studies showing a 20–25% *radiographic* response rate, but unclear survival benefit following bevacizumab therapy (92, 93). Stemming from the work of Judah Folkman and others in the 1980s, who detailed the mechanisms and importance of angiogenesis in cancer (94, 95), bevacizumab targets vascular endothelial growth factor (VEGF) using a humanized monoclonal antibody. Specific blockade of VEGF effectively decreases the growth of new blood vessels into growing tumors in pre-clinical studies, a key feature of MG (96). Bevacizumab is an IV infusion and, thus, is subject to the limitations imposed by both the BBB and BPB. As a monoclonal antibody, the adhesive characteristics (97), and the size (~10 nm), of this molecule may strongly hinder both the transport across the BBB in more normal brain areas with infiltrating tumor cells, as well as movement through the ECS and eventual intraparenchymal distribution. More recently, direct interarterial (IA) delivery of bevacizumab with local BBB disruption has been investigated to overcome these limitations (98, 99), however, the survival benefit of this approach is pending.

In clinical practice, bevacizumab appears to markedly reduce cerebral edema and likely modulates the BBB, but has an unclear effect on the patho-biology of MG (100–102). In two randomized, placebo-controlled clinical trials assessing if the addition of bevacizumab to standard chemoradiation therapies would improve survival in patients with newly diagnosed GBM, bevacizumab was found to improve progression-free but not overall survival (103, 104). Bevacizumab is emerging as a steroid-sparing agent for MG patients with significant tumor-associated edema suffering from the side-effects of long-term steroid use. Of note, there is concern that anti-VEGF treatment may transiently improve the radiographic appearance, but may veil or even worsen the underlying disease. While there is not direct evidence that isolated VEGF inhibition leads to upregulation or activation of more pathogenetic tumor pathways, the reports of rapid disease progression following bevacizumab monotherapy (100, 101) highlight this possibility and the need to focus on combination treatments regimens, as discussed earlier.

Together these successes and failures along the path to the current treatment standards reveal some key considerations in designing effective delivery strategies and clinical trials for MG. First, novel trial designs will need to be considered that allow concurrent evaluation of agent combinations, in the context of current therapies known to modulate the BBB and immune system (steroids, radiation, bevacizumab). Second, agents that have shown powerful effects against tumor cells *in vitro*, will likely need to be coupled with thoughtful delivery strategies to increase chances of achieving *in vivo* efficacy – especially given the size difference between most pre-clinical models (mice, rats) and humans. In summary, important delivery considerations include: effective transport across the BBB, enabling enhanced movement through brain and tumor tissue to achieve adequate drug distribution in the regions of infiltrating tumor cells, and providing sustained, multi-modal actions against that specific patient’s tumor cells.

## Examples and Opportunities for New Therapeutic Strategies

### Prospects for systemic delivery

Early studies exploring systemic delivery of drugs and drug-loaded NPs aimed to capitalize on passive accumulation of these agents in the tumor due to the EPR effect. The EPR effect suggests that drugs and particles may accumulate in the tumor core due to leaky blood vessel and in some cases, long circulation times (e.g., some particles and antibodies), but helps little in brain regions where neovascularization and tissue remodeling have yet to begin (105, 106). Favel et al. studied systemically administered liposomal doxorubicin in a Phase II trial of MG patients and observed this treatment led to disease stabilization in 54% and suggested prolonged survival compared to historical controls (107). Other studies have suggested that NP formulations of some drugs may aid in the delivery across the BBB (108, 109), possibly via LDL-receptor or other endocytic pathways. Yet, to increase the portion of the total IV load making it to the tumor, additional strategies have been proposed to navigate across the BBB.

### Intravascular delivery with BBB disruption

**Figure 3:**
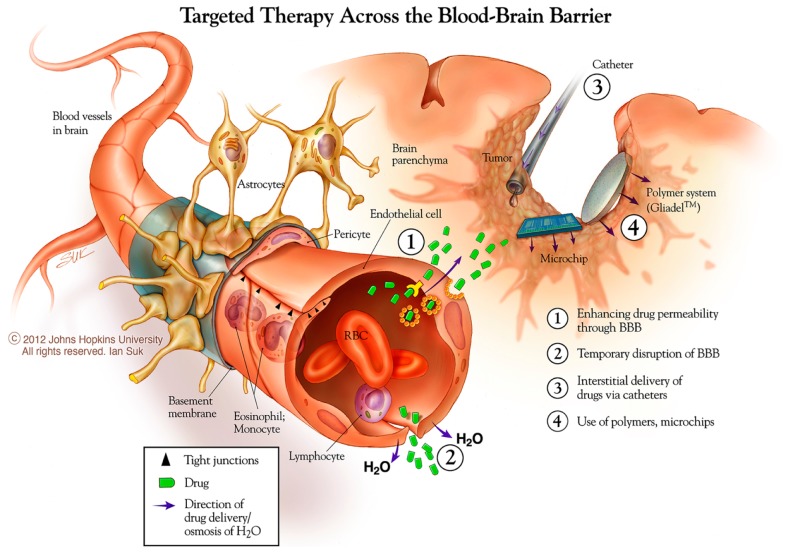
**New approaches to brain tumor therapies**. Some possibilities for this include enhancing drug permeability across the blood brain barrier/neurovascular unit, including temporary disruption of this interface using chemical (mannitol) and physical (ultrasound) means. The distribution of therapies may be enhanced using catheter-based convection enhanced approaches. Biodegradable polymer wafer, particle, and microchip reservoir systems are being explored further for timed and/or sustained release of drugs, as well as targeting tumor-specific structures. *Copyright Ian Suk 2012 – Johns Hopkins University.

One strategy for systemic drug delivery to brain tumors involves bypassing the BBB via mechanical or chemical disruption (Figure 3). A promising approach uses magnetic resonance (MR)-guided focused ultrasound (MRgFUS) with intravenous microbubbles (MB) to locally and specifically disrupt the BBB and improve the accumulation of drug and/or NPs from blood into the sonicated region (110). The sonication parameters can be tuned to provide both reversible (drug/particulate delivery alone) or irreversible (drug/particulate delivery plus tissue damage) BBB opening in a conformal region defined by the MRI data. Therapeutic agents can be loaded into particles with and without direct conjugation to or encapsulation within the MB (111–113). This minimally invasive, non-surgical approach may also be useful for unresectable and recurrent/residual brain tumors not amenable to surgery. Clinical trials are currently being planned for MG using the MRgFUS with drug-loaded NP approach.

Blood brain barrier disruption may also be achieved using osmotic agents (114) (e.g., mannitol), pro-inflammatory cytokines (e.g., IL-17) (115, 116), or blood vessel modulators (e.g., RMP-7) (117). A Phase II trial comparing the combination of BBB disruption using RMP-7 and the chemotherapy carboplatin to the chemotherapy alone, showed minimal improvement in the time to progression or survival in patients with MG (118). While these are promising approaches to circumventing the BBB, once across, the drugs and/or particles would still need to penetrate and distribute within the brain parenchyma, and ideally have sustained action within tumor tissue, to have a meaningful effect.

### Intravascular delivery with BBB shuttling or targeting

The goal of many BBB transport studies has been identifying a specific receptor or endothelial surface component to enable high-efficiency, non-degradative transcytosis of drugs and delivery vehicles from the blood into the CNS. The concept of the “molecular Trojan horse” has been suggested for disguising therapeutic moieties with endogenous molecules known to initiate receptor-mediated transcytosis (119). Initially, promising candidates for this included the transferrin (Tf) receptor (120) and lipoprotein receptor-related protein-1 (LRP-1) (121) as well as specialized particle surface coatings such as polysorbate 80 (109). While tagging drugs and delivery vehicles with ligands or monoclonal antibodies for these cell membrane proteins showed some promising results (120, 122, 123), the fraction of the total IV load that reaches the brain is still low (124, 125). Early-phase clinical trials are underway for systemic delivery of Tf and LRP-1 conjugates for brain tumors, but efficacy results are pending. One particularly exciting approach has been to decrease the affinity of the targeting moiety for its ligand, which then can increase the BBB transcytosis and release of therapeutic entities into the brain (123).

### Biologic strategies

The promise of genetically re-programing key pathways gone awry in cancer cells and tumor micro-environment, as well as engineering microbes to seek and destroy these cells, has led to the development of numerous viral- and bacterial-based treatment systems. Multi-modal effects may also be possible using microbial-based delivery strategies since, theoretically, they can be engineered to deliver numerous therapeutic agents. The potential for prolonged survival of transfected cells and sustained transgene expression are also possible benefits of this approach.

Many viruses have been investigated for therapeutic delivery to, or direct destruction of, brain tumors. Notable examples include herpes simplex virus (HSV) (126), vesicular stomatitis virus (VSV) (127), retrovirus (RV) (128), adenovirus (AV) (129), and adeno-associated virus (AAV) (130). Each of these can be selected for specific tropism, replication properties, or surface capsid characteristics, thereby promoting cell targeting, virion distribution, or intracellular effects. A good example of this was described in the study by Ozduman et al. where a replication competent, MG-adapted VZV strain was selected and found to bind, enter, and kill MG cells *in vivo* (127). Limitations to this approach include the potential for immunogenicity, leading to an adaptive immune response to the virus and subsequent inflammation and cerebral edema that can be deadly. In addition, this host response can limit repeat dosing. Further limitations include the ability to precisely control viral replication and/or transgene production. In part due to these potential problems, few Phase III clinical trials using viral-based gene therapy have been performed.

In 2000, Rainov and colleagues reported the results of a multi-center Phase III randomized, controlled trial of fibroblast-transfected, RV-mediated delivery of herpes simplex virus thymidine kinase (HSV-tk) in patients with untreated MG. They found no difference in median survival between the two treatment groups: standard therapy (surgery plus radiation) versus standard therapy with adjuvant gene therapy delivered via direct intraparenchymal injection during surgery (128). While this study demonstrated the feasibility of this local, cell-based, gene therapy approach, the authors suggested the lack of efficacy was due to poor distribution of the transfected, non-migratory fibroblast cells and subsequently limited delivery of the HSV-tk gene product to the tumor cells (128). Movement of the virus or the end-effector (HSV-tk), whether a transfected cell or transgene product, is more complicated in this case, involving both active movement and passive diffusion of the therapeutic components. Of note, all of the viruses mentioned above except for AAV, are larger than 100 nm (significantly bigger than the reported size limit), therefore diffusion through the brain parenchyma is expected to be limited. Active movement of virions transported within intrinsic cells or via transfected cells through the brain will also alter the distribution of these delivery agents. The analysis from the Rainov study authors, based on the strong pre-clinical data showing excellent efficacy (131), suggests that distribution is still a crucial limiting factor that must be addressed for viral and cell-based therapies in humans. Lang and colleagues have offered a potential solution to this problem by using mesenchymal stem cells to deliver oncolytic viruses systemically. Pre-clinical evidence suggests this may be a promising delivery modality (132, 133).

Another interesting microbial delivery strategy involves using anaerobic bacterial spores. IV-injected *Clostridium novyi* spores have been shown to germinate within the avascular, hypoxic regions of tumors and destroy surrounding viable tumor cells (134). In addition, these spores appear to stimulate a potent anti-tumor immune response, which has the potential to eliminate infiltrating cells in normoxic tissue (135). Clinical trials are being planned around this technology. Clearly, the possibility of generating a sustained, specific anti-tumor immune response would go a long way to addressing the delivery limitations as the immune cells would be able to actively seek the target cells, and destroy cells based on the antigenic differences. This concept will be discussed further in the next section.

### Cell-based approaches

Given the therapeutic potential for eliciting a potent anti-tumor immune response, another attractive delivery strategy has been to stimulate autologous antigen-presenting cells (APCs) to activate cytotoxic and helper T-cells to recognize and eliminate tumor cells in the CNS. APCs can be harvested from the patient’s peripheral blood, pulsed with tumor lysate stimulated with cytokines, or transfected with a desired transgene (136, 137). In two Phase I trials using tumor lysate-pulsed autologous peripheral blood dendritic cells for patients with MG, there were no adverse reactions and about half of the patients demonstrated specific adaptive immune responses to the tumor antigens (137, 138). In the Phase II study, a correlation was found between vaccine-responders, those who developed tumor-specific cytotoxic T-cell responses post-vaccination, and time to progression and survival (139). Ongoing work in this area, including three phase III clinical trials, is focused on identifying the specific components that enhance the anti-MG immune response by modulating the tumor micro-environment and limiting immune tolerance (140–142).

Another cellular strategy has been to capitalize on the observation that autologous stem cells, derived from embryonic or mesenchymal cell lineages, appear to target to and accumulate in brain tumors (143–145). Although no clinical trials have been performed in this area to date, these cell-based delivery strategies offer unique possibilities. Stem and immune cells have the innate ability to move within the body and tissues. Active trafficking across endothelial surfaces and within the ECS of tissues are hallmarks of immune cells, thereby enabling recruitment to the specific sites, be it infection, inflammation, tissue repair, or tumor modulation. Genetically engineered cells or activated cytotoxic immune cells, offer the potential for multiple anti-tumor effects mediated by cytokines and pro-apoptotic agents (146–150).

### Intra-cerebro-spinal fluid delivery

Following intra-CSF administration, the concentration of many drugs and molecules in the brain parenchyma has been found to be negligible (151). For this reason, treatment of intrinsic CNS tumors with chemotherapy administered into the CSF has not yet been proven effective. However, using the intra-cerebro-spinal fluid (ICSF) route of administration for drug delivery to the brain has proven successful in other conditions where CNS tissue penetration is less critical, such as meningeal carcinomatosis, spasticity, chronic pain, and lymphomatous meningitis. Intrathecal baclofen is used to treat spasticity (152), intrathecal opioids are used to treat chronic pain (153), and intrathecal chemotherapy for meningeal carcinomatosis (154) and lymphoma (155). Importantly, in most cases, the intrathecal/intraventricular approach has delivered the drugs close to ventricular surfaces. The 150 ml average volume of CSF in the human CNS is completely turned over every 6–8 h, and exits the brain mainly into the blood. Moreover, ICSF drug delivery to the brain results in high drug exposure at the ependymal surface of the brain, which can cause a subependymal inflammatory reaction and tissue damage (155). A paradox of ICSF drug administration is that in many cases, the drug distributes to the blood much better than it does to the brain due to this rapid circulation and clearance pathway (10). As such, an ICSF injection is more similar to a slow IV injection rather than a direct intraparenchymal injection for many drugs (151).

### Intranasal delivery

Intranasal administration of various medications and drug-loaded NPs has been studied and suggested as a means of near-direct delivery to the CNS via olfactory neurons within the nasal mucosa (121). Hormones (e.g., vasopressin, calcitonin) delivered via nasal sprays are perhaps the best-studied and widely used intranasal agents aimed at CNS effects. A recent observational trial of the chemotherapeutic, perillyl alcohol, delivered intranasally in patients with recurrent MGs showed minimal toxicity but no direct evidence of CNS drug levels or anti-tumor activity (156). While this approach may bypass the limitations of the BBB and be useful for agents that exert effects at low dosages, controlling larger drug or particle doses and distribution represents a major limitation in the treatment of larger or more specific brain regions, as would be the case for many brain tumors.

### Examples and opportunities using direct, local delivery

Direct local delivery, particularly at the time of surgery for tumor biopsy or removal, offers a unique access opportunity to bypass one of the three delivery barriers, the BBB. Two major strategies have been used for direct CNS delivery in the clinical arena: drug-loaded biodegradable polymer systems and catheter-based CED (Figure 3). In addition, non-surgical approaches for systemic delivery across the BBB, either by enhanced permeability or improved trafficking, offer the benefits of non-invasive, systemic administration with the potential that a larger portion of the total dose will reach the desired target. In all, these strategies offer the capability to increase the maximum tolerated dose of a drug by avoiding systemic side-effects, and improving drug distribution in the brain and peri-tumoral region.

With the first description of controlled-release polymers for delivery of macromolecules in 1976 (157), a new field and industry developed around the concept of local drug delivery for various human conditions (158). The evolution of this technology led to the need for biodegradable, implantable systems that would provide the desired therapeutic effect without the requirement of removal. The biodegradable poly-anhydride polymers, including poly[bis(*p*-carboxyphenoxy)propane-co-sebacic acid] eventually used in Gliadel^®^, helped to solve this problem and created a platform for clinical translation (159). Therapeutic agents could be encapsulated within or formulated with these polymers to provide the desired drug loading and release kinetics. The encapsulation of drugs into polymeric delivery systems offers numerous potential advantages over the delivery of free drug alone. These include: protection from clearance and degradation mechanisms, tuning of the drug loading and sustained release profile, and improved efficacy and reduced toxicity of a given amount of the drug (160). Further advances in polymer technology have come in the development of new materials (e.g., fatty-acid dimer–sebacic acid (FAD–SA) and poly (lactic co-glycolic acid) (PLGA) with unique physico-chemical properties that enable encapsulation of a broad spectrum of compounds and macromolecules (161, 162).

The next clinical evolution in therapeutic delivery for brain tumors involved an expandable balloon catheter that is placed in the resection cavity at the time of tumor debulking [GliaSite^®^ Radiation Therapy System (RTS), Cytyc Surgical Products, Palo Alto, CA, USA]. Approximately 2–4 weeks after surgery, the balloon is filled with a radioactive aqueous solution [Iotrex (sodium 3-(125 I)-iodo-4-hydroxybenzene sulfonate)] for a predetermined amount of time, during which a therapeutic dose of radiation is delivered to the margin of the surgical cavity. After completion of the calculated dwell time, the solution is removed and the balloon catheter is retrieved transcutaneously. While this approach has been shown to be feasible and safe (163), a clear survival advantage has not been shown (164, 165).

The next step included CED through implanted intracerebral catheters, which offers the potential advantage of better drug distribution to distant invading cancer cells compared to other strategies aided only by passive diffusion. Challenges to this approach have included side-effects caused by backflow along the catheter often due to high interstitial pressure, drug leakage in non-desired regions, inclusion of contrast visualization agents, and poor/unequal distribution of delivered agents. Combining CED with drug-loaded particle systems has been investigated to overcome these problems. Allard et al. described the ideal CED nanocarrier as about 20–50 nm in size, with a global neutral or negative charge, and shielded by a steric coating made of PEG or dextran (87). Our recent study suggests that even larger particles may be used, if appropriately coated to minimize adhesive interactions (40).

### Particle-based systems

Microspheres and NPs of various forms and compositions have proven useful in formulating diagnostic and therapeutic agents for local, as well as systemic delivery to brain tumors (166, 167). One of the unique aspects of biomaterials-based strategies is the flexibility to pair the drug with an appropriate formulation material to achieve the desired drug loading and/or release kinetics. In addition, small semi-conductor and metal particles [a.k.a. quantum dots (QDs)] offer unique optical and electronic properties for multi-spectral imaging as well as the potential to introduce thermal effects within tumors and cells. QDs are an example of the versatility of these particle platforms for designing multifunctional imaging and therapeutic delivery systems (168–170).

In several recent publications, relevant pre-clinical and clinical trials, as well as laboratory studies using particle-based therapeutics for CNS disease and brain tumors were reviewed (87, 171, 172). The characteristics important for movement of these particulate systems in the brain include size (~100 nm) and surface chemistry (steric coating of PEG or dextrans), have been highlighted (40, 87). Researchers are beginning to use these criteria to design various particle systems for brain cancer treatment, including liposomes (107, 173, 174), poly(lactic) acid (PLA) NPs and poly(lactic-co-glycolic acid) (PLGA) NPs (175), and dendrimer NP’s (176) to name a few. One of the best-studied drugs with NP formulation for MG is doxorubicin (DX). DX is a chemotherapeutic agent that intercalates into cellular DNA, leading to cell death. Systemic use of DX is limited by cardiac and liver toxicity, hence the need for additional formulations and delivery strategies. A Phase I/II trial of liposomal DX in MG patients suggested disease stabilization with minimal treatment-related toxicity (107). While no clear candidates have emerged to advance to the level of a Phase III clinical trial, other liposomal systems have shown some promise (107).

A central feature of particle engineering is that the surface coating can be modified to optimize transport and/or targeting properties. This makes it feasible to design particles that can access and potentially target to infiltrating cancer cells through tissue penetration within the ECS. To realize this goal, the maximum size and ideal surface characteristics for particulate delivery systems needs to be closely defined; recent work by our group using high resolution microscopy with multiple particle tracking techniques has further delineated these parameters (40). NPs as large as 114 nm in diameter diffused within the human and rat brain tissue, but only if they were densely coated with poly(ethylene glycol) (PEG). Based on the movement of these particles, we estimated that human brain tissue ECS has some pores larger than 200 nm and that more than one-quarter of all pores are at least 100 nm.

The ability to engineer larger drug-loaded particles with maximal safe drug loading and optimized release kinetics make this biomaterials approach one of the more promising opportunities for effective drug delivery. Combining multiple therapeutic agents in each particle, or combining multiple types of particles loaded with different agents (drugs, plasmids, inhibitory oligonucleotides, etc.) are also possibilities for addressing the need for combination therapy.

### Microreservoir drug-loaded arrays

Biodegradable polymers have been used to fabricate drug-loaded microreservoir arrays (MicroChips^®^) that release drugs from numerous small reservoirs in an actively controlled fashion (Figure 3) (177). The timing of release is based on the rupture of nitride membranes covering the drug reservoirs, controlled by a computer-based, wireless programing device. This pharmacy-on-a-chip approach has shown efficacy against pre-clinical *in vivo* brain tumor models using timed release of BCNU and TMZ chemotherapies (178, 179). The first human testing of this device has been performed, demonstrating safety and physiologically relevant, pulsatile release of parathyroid hormone for the treatment of osteoporosis (180). While still preliminary, these studies suggest a promising role for this technology in designing local, sustained release treatments that can be adapted in a multi-modal fashion over time.

## Conclusion

Important delivery considerations for effective therapies for MG include: effective transport across the BBB, enabling enhanced movement through brain and tumor tissue to achieve adequate drug distribution in the regions of infiltrating tumor cells, and providing sustained, multi-modal actions against that specific subclass of tumor cells. By considering these key barriers, and focusing on the residual infiltrating cancer cells, major improvements in the outcomes for MG patients may be realized. Advances in therapeutic delivery methods raise hope that the increasing understanding of brain tumor patho-biology, including genetics and epigenetics, will lead to promising new therapies for this devastating disease.

## Conflict of Interest Statement

The authors declare that the research was conducted in the absence of any commercial or financial relationships that could be construed as a potential conflict of interest.
